# Rescuing Fertilization Failure in ICSI: A Narrative Review of Calcium Ionophore Activation, PLCζ Testing, and Embryo Morphokinetics

**DOI:** 10.3390/biomedicines13082007

**Published:** 2025-08-18

**Authors:** Charalampos Voros, Despoina Mavrogianni, Diamantis Athanasiou, Ioakeim Sapantzoglou, Kyriakos Bananis, Antonia Athanasiou, Aikaterini Athanasiou, Georgios Papadimas, Charalampos Tsimpoukelis, Ioannis Papapanagiotou, Dimitrios Vaitsis, Aristotelis-Marios Koulakmanidis, Maria Anastasia Daskalaki, Vasileios Topalis, Nikolaos Thomakos, Marianna Theodora, Panagiotis Antsaklis, Fotios Chatzinikolaou, Dimitrios Loutradis, Georgios Daskalakis

**Affiliations:** 1 Department of Obstetrics and Gynecology, ‘Alexandra’ General Hospital, National and Kapodistrian University of Athens, 80 Vasilissis Sofias Avenue, 11528 Athens, Greece; depy.mavrogianni@yahoo.com (D.M.); kimsap1990@hotmail.com (I.S.); tsimpoukelischa@gmail.com (C.T.); aristoteliskoulak@gmail.com (A.-M.K.); md181341@students.euc.ac.cy (M.A.D.); thomakir@hotmail.com (N.T.); martheodr@gmail.com (M.T.); panosant@gmail.com (P.A.); gdaskalakis@yahoo.com (G.D.); 2 IVF Athens Reproduction Center, 15123 Maroussi, Greece; diamathan16@gmail.com (D.A.); antoathan16@gmail.com (A.A.); diamathan17@gmail.com (A.A.); 3 King’s College Hospitals NHS Foundation Trust, London SE5 9RS, UK; kyriakos.bananis@nhs.net; 4 Athens Medical School, National and Kapodistrian University of Athens, 15772 Athens, Greece; dr.georgepapadimas@gmail.com (G.P.); gpapamd@hotmail.com (I.P.); vaitsisdim@gmail.com (D.V.); vtopalismd@gmail.com (V.T.); loutradi@otenet.gr (D.L.); 5 Laboratory of Forensic Medicine and Toxicology, School of Medicine, Aristotle University of Thessaloniki, 54124 Thessaloniki, Greece; fotischatzin@auth.gr; 6 Fertility Institute-Assisted Reproduction Unit, Paster 15, 11528 Athens, Greece

**Keywords:** artificial oocyte activation, intracytoplasmic sperm injection, phospholipase C zeta, calcium ionophore, oocyte activation deficiency, embryo morphokinetics, time-lapse imaging, fertilisation failure, calcium signalling, assisted reproductive technology

## Abstract

Fertilisation failure following intracytoplasmic sperm injection (ICSI) is a significant challenge in assisted reproductive technology (ART), particularly in the absence of an identifiable cause. Artificial oocyte activation (AOA), typically with calcium ionophores, has emerged as a potential solution in scenarios characterised by a deficiency of phospholipase C zeta (PLCζ). This narrative review consolidates the latest clinical and experimental data regarding the application of calcium ionophores for oocyte activation, the significance of PLCζ testing in instances of unexplained fertilisation failure, and the impact of AOA on the morphokinetics and developmental potential of embryos. AOA has demonstrated an enhancement in fertilisation, cleavage, and pregnancy outcomes in specific patient populations, including individuals with diminished ovarian reserve or those who have previously attempted conception unsuccessfully. Although AOA appears to have no impact on embryo morphokinetics, certain studies indicate slight alterations in early cleavage features. The available statistics indicate that there are no significant safety concerns about outcomes for babies. This finding underscores the significance of tailored ART methodologies that incorporate molecular diagnostics and targeted AOA therapies. It emphasises the necessity for additional prospective trials to enhance patient selection and long-term safety surveillance.

## 1. Introduction

ICSI has revolutionised ART by enabling successful fertilisation for individuals with significant male factor infertility and other conditions. Notwithstanding the elevated success rates and extensive applicability of ICSI, a significant proportion of couples persist in encountering unexplained fertilisation failure or inadequate fertilisation rates post-procedure, despite the lack of discernible anomalies in gamete quality or laboratory conditions [1,2]. Partial fertilisation failure (fertilisation <30%) is noted in up to 25% of cycles, but TFF occurs in roughly 1–3% of ICSI cycles. This imposes considerable emotional and financial strains on the affected couples [3]. In mammals, fertilisation is a meticulously regulated biochemical cascade that transcends the mere mechanical penetration of the sperm into the oocyte. Oocyte activation is a vital and irreversible process that initiates embryonic development. It is a sequence of synchronised biochemical and cellular alterations that enable the oocyte to complete meiosis and initiate development. The primary factor inducing this activation is the generation of recurrent calcium (Ca^2+^) oscillations within cells, which serve as the principal signal for subsequent activities [4].

PLCζ, a soluble component exclusive to sperm, initiates calcium oscillations following the fusion of sperm and egg cells. PLCζ is exclusively located in the vicinity of the sperm head nucleus, and upon membrane fusion, it is discharged into the ooplasm. It does not utilise receptor-mediated pathways or the conventional G-protein coupled mechanisms observed in somatic cells [5,6]. Upon entry into the oocyte, PLCζ exhibits significant activity in hydrolysing phosphatidylinositol PIP2, located on the inner leaflet of the oolemma and within cytoplasmic vesicles. This hydrolysis generates two significant second messengers: diacylglycerol (DAG) and inositol 1,4,5-trisphosphate (IP_3_). IP_3_ rapidly binds to IP_3_R1, which are abundantly located on the endoplasmic reticulum (ER), the primary site for calcium storage within oocytes [7,8]. The alteration in the conformation of the IP_3_ receptor induces the release of Ca^2+^ into the cytosol in bursts, manifesting as oscillations that may persist for hours in human oocytes. These oscillations possess a certain periodicity, amplitude, and spatial localisation, all of which are crucial for effectively activating the egg.

Increased cytosolic Ca^2+^ levels activate calmodulin-dependent protein kinase II (CaMKII), which subsequently phosphorylates and activates the anaphase-promoting complex/cyclosome (APC/C). The APC/C is a ubiquitin ligase that designates cyclin B for degradation by the proteasome [9]. This degradation diminishes the activity of cyclin-dependent kinase 1 (CDK1), hence inhibiting the function of the maturation-promoting factor (MPF). MPF is a complex that maintains the oocyte in metaphase II arrest. Upon deactivation of MPF, the oocyte is able to exit meiotic arrest and proceed with growth [10,11].

Simultaneously, Ca^2+^ oscillations facilitate cortical granule exocytosis, altering the zona pellucida to prevent polyspermy and ensure fertilisation occurs with a single sperm. The synthesis of DAG subsequently activates calcium-dependent effectors, particularly protein kinase C (PKC) isoforms, which are essential for reorganising the oocyte’s cytoskeleton and cytoplasmic structure—processes critical for cortical granule exocytosis, zona pellucida alteration, and successful zygote development [12]. It is essential that these Ca^2+^ oscillations are both present and precise in terms of timing and spatial distribution. Alterations in oscillation patterns, such as reduced frequency or delayed onset, have been associated with fertilisation failures, suboptimal embryo development, and complications in blastocyst formation [13]. In assisted reproduction, ICSI is particularly significant as it can physically inject sperm, potentially bypassing or failing to initiate the physiological signalling cascade, especially in cases of PLC deficit or mutation [2]. The PLCζ-IP_3_-Ca^2+^ pathway for oocyte activation offers a very specific mechanism for sperm to transmit signals to the maternal cytoplasmic machinery. It is the molecular pivot that dictates the success of fertilisation and, consequently, the initiation of life. Numerous unexplained fertilisation failures in ART are attributable to issues with this axis, which may be genetic, structural, or functional anomalies in sperm. This underscores the necessity for tailored interventions such as AOA [8,14].

## 2. Oocyte Activation Deficiency (OAD) and the Role of PLCζ in Fertilisation Failure

Oocyte activation is a vital molecular event in the fertilisation process. This is the moment when the arrested oocyte resumes meiosis and initiates embryonic development. In mammals, oocyte activation is primarily initiated by a biochemical signalling cascade facilitated by sperm-derived proteins, such as PLCζ, rather than solely by the physical entry of sperm into the egg cytoplasm [8,14]. The phenomenon occurs due to a specialised and dynamic biochemical signalling cascade initiated by the sperm-specific PLCζ. This enzyme belongs to the phospholipase C family, although it is exclusively present in mature spermatozoa. When the gametes merge, PLCζ is released into the cytoplasm of the oocyte [5]. It initiates the degradation of PIP_2_, a membrane-bound phosphoinositide, into two secondary messengers: IP_3_ and DAG. IP_3_ is crucial since it interacts with IP_3_Rs, predominantly located on the ER membrane. This results in the rapid and prolonged release of Ca^2+^ into the cytoplasm [15,16].

This mechanism induces calcium oscillations within cells that recur repeatedly, typically commencing a few minutes post-sperm entry and persisting for several hours. The Ca^2+^ oscillations occur systematically; they are meticulously regulated and essential for subsequent activation pathways [17]. These oscillations activate a complex molecular network, notably CaMKII, which phosphorylates and inactivates early mitotic inhibitor 2 (Emi2). This halts the cell cycle at metaphase II and initiates the anaphase-promoting complex/cyclosome (APC/C). Upon activation of APC/C, it conjugates ubiquitin to cyclin B, facilitating its degradation [18,19]. This inhibits CDK1, a crucial component of the maturation-promoting factor (MPF). The reduction in MPF activity permits the oocyte to exit metaphase II, complete meiosis, and initiate pronuclear formation and early zygotic development [20].

The disruption of this PLCζ-dependent signalling pathway results in a condition known as OAD. OAD represents a molecular-level failure of fertilisation. Inadequate or missing calcium oscillations prevent the egg from completing meiosis, halting it at the metaphase II stage and leading to fertilisation failure. The predominant and clinically significant aetiology of OAD is the dysfunction or absence of PLCζ [10,21]. Molecular dysfunction can occur in sperm that appears and operates normally based on routine semen examination, making this significant. This illustrates the limitations of conventional sperm assessment and underscores the necessity of advancing beyond morphological and quantitative metrics to functional and molecular diagnostics [22].

PLC deficiency may manifest in various ways at the molecular level. In certain individuals, the PLCZ1 gene functions improperly, resulting in the absence of PLCζ protein synthesis. In specific instances, genetic alterations result in the production of structurally adequate proteins that are incapable of hydrolysing PIP_2_ [23]. Men experiencing total fertilisation failure (TFF) exhibit missense mutations in critical regions of PLCζ, specifically within the EF-hand, XY catalytic domains, and C2 domain [24]. For instance, variants such as H398P and I489F have been shown to diminish enzyme efficacy without altering protein stability or localisation. In certain cases, PLCζ is produced but mislocalised within the sperm cell, typically confined to the midpiece or tail, hence hindering its proper transfer into the egg cytoplasm during fertilisation [25]. If the sperm cytoskeleton fails to anchor the enzyme to the postacrosomal sheath, which is believed to be crucial for efficient delivery, or if the post-translational modification is improperly executed, the enzyme may localise incorrectly [26].

According to these discoveries, “molecular infertility” refers to a clinical phenotype characterised by fertilisation failure due to issues with molecular signalling proteins, rather than any apparent abnormalities in sperm morphology, motility, or concentration. PLCζ exemplifies this effectively. Researchers consistently demonstrate that sperm from men with idiopathic infertility or specific conditions, such as globozoospermia, lack functionally localised PLCζ and fail to induce Ca^2+^ oscillations, even when directly injected into oocytes using ICSI [8,27].

Due to its critical role in oocyte activation, much research is currently being conducted to identify PLCζ in clinical settings. Immunocytochemistry (ICC), utilising monoclonal or polyclonal antibodies targeting PLCζ, has demonstrated efficacy in detecting PLCζ within preserved sperm samples and localising its cellular distribution [8,24]. A functional PLCζ is often located in the perinuclear theca and the equatorial region of the sperm head [27]. Conversely, mislocalised or undetectable PLCζ is strongly associated with TFF following ICSI. Additional quantitative techniques, such as Western blotting and RT-PCR, can validate expression at the protein or mRNA level; however, they lack spatial resolution and are challenging to implement in most ART laboratories [28].

Despite initial studies indicating acceptable results, numerous challenges persist in standardising PLCζ testing and its clinical application. Currently, there is no commercially available assay kit that includes validated criteria or assurances of reproducibility. The variability in fixation protocols, antibody specificity, staining techniques, and subjective interpretation complicates clinical use. PLCζ screening remains classified as experimental in the majority of ART environments due to the absence of defined methodologies and proven diagnostic criteria. Nonetheless, its clinical utility is being recognised as additional evidence emerges indicating its efficacy in identifying oocyte activation deficiencies [29].

Nonetheless, the identification of PLCζ has purposes beyond mere diagnostics. Physicians can employ a precision medicine strategy to adjust medications, as they are now able to identify sperm with PLCζ dysfunction [30]. Patients identified with PLC deficiency may benefit from AOA using calcium ionophores, which elevate intracellular Ca^2+^ levels and render PLC redundant. This technique has been shown to reinstate the capacity to fertilise ova and accelerate embryonic development in instances of OAD [30,31]. This demonstrates that PLCζ testing is functionally significant. Examining sperm for PLCζ expression may assist couples facing infertility due to factors unrelated to sperm, such as oocyte cytoplasmic immaturity or mechanical zona hardening, in avoiding unnecessary or repeated ICSI cycles [32].

Recent findings indicate that PLC-mediated calcium oscillations may significantly influence development, potentially impacting zygotic genome activation, chromatin remodelling, and epigenetic programming. The recent discoveries underscore the necessity of identifying and rectifying PLCζ-related issues, not just for fertilisation but also for optimal embryonic development and sustained reproductive success [33,34].

In conclusion, the absence of oocyte activation resulting from PLCζ dysfunction represents a definitive molecular mechanism that leads to fertilisation failure in assisted reproduction. Recent advancements in molecular biology have elucidated the mechanisms of signalling networks and demonstrated the inadequacies of conventional semen analysis in detecting infertility. Functional biomarkers such as PLCζ will undoubtedly play a role in the future of reproductive medicine. They will facilitate personalised therapy and enhance outcomes for those experiencing unexplained infertility. To completely actualise this promise, it is essential to establish standardised diagnostic platforms, conduct multicentre validation studies, and develop regulatory frameworks that facilitate the integration of PLCζ testing into routine clinical practice.

Recent advancements in molecular reproductive medicine have identified many PLCζ anomalies that result in unexplained fertilisation failure following ICSI. Table 1 categorises these problems into four primary classes. Mutations that inhibit the functionality of the PLCZ1 gene typically result in the full absence of the PLCζ protein. This indicates that the oocyte cytoplasm is incapable of initiating calcium oscillations. This type is most probable to induce TFF and can be detected using Western blotting or ICC. Secondly, missense mutations, particularly those affecting the EF-hand, catalytic XY domains, or C2 domain of PLCζ, might produce proteins with identical structures that exhibit less enzymatic functionality. Mutations such as H398P and I489F may permit protein synthesis and precise localisation; nevertheless, they will be incapable of hydrolysing PIP_2_ to produce IP_3_, resulting in OAD. Third, in certain sperm samples, PLCζ is produced and physically intact, although it is mislocalised within the sperm cell. For instance, it may reside in the midpiece or tail rather than the postacrosomal sheath of the sperm head. This misplacement complicates the sperm’s journey to the egg during fertilisation, resulting in a lack of activation despite adequate sperm parameters. The utilisation of anti-PLCζ antibodies in immunocytochemistry is the most dependable method for identifying such spatial anomalies. Ultimately, there are instances when PLCζ is produced at standard levels yet fails to function due to minor post-translational modifications or undetected functional defects. These circumstances are challenging to identify, and model oocytes generally require both immunocytochemistry and functional activation assessments for confirmation.

## 3. Artificial Oocyte Activation: Molecular Mechanism and Clinical Application

Comprehending the initiation of embryogenesis necessitates a precise understanding of the intracellular calcium signalling cascade. This forms the foundation for the concept of AOA. The egg remains arrested in metaphase II of meiosis until it receives a unique molecular signal, characterised by a sequence of cytoplasmic Ca^2+^ oscillations initiated by the introduction of sperm-specific PLCζ [4]. The meticulously orchestrated release of Ca^2+^ from endoplasmic reticulum reserves initiates a sequence of precisely regulated processes, encompassing the inactivation of MPF, the reinitiation of meiosis, the exocytosis of cortical granules, and the development of pronuclei. If this calcium signalling pathway fails, fertilisation ceases at the stage of oocyte activation, clinically referred to as OAD [35,36].

Artificial oocyte activation is a methodologically engineered intervention designed to replicate the critical calcium influx typically initiated by PLCζ. Calcium ionophores, such as ionomycin and A23187, are the predominant agents utilised in AOA. They facilitate the direct translocation of extracellular Ca^2+^ across the oolemma into the egg cytoplasm [37]. These medicines function by transiently disrupting the lipid bilayer, facilitating the rapid influx of calcium without necessitating IP_3_ synthesis or receptor-mediated endoplasmic reticulum signalling [38]. This approach lacks the physiological, rhythmic characteristics of endogenous PLCζ-mediated activation; yet, it suffices to initiate the molecular processes required for exiting meiosis and advancing the oocyte towards zygotic development [39].

The artificial Ca^2+^ spike activates CaMKII at the molecular level. The enzyme then attaches a phosphate group to EMI2. This induces the breakdown of EMI2, activating the anaphase-promoting complex/cyclosome (APC/C), a ubiquitin ligase complex that designates cyclin B1 for proteasomal degradation [40]. The degradation of cyclin B1 inhibits CDK1, an essential component of the MPF complex. This liberates the oocyte from its arrest in metaphase II. Concurrently, the spindle apparatus reorganises, the second polar body is expelled, and the microtubules undergo depolymerisation. These indicators signify that the oocyte has been effectively triggered [41,42].

The effectiveness of artificial oocyte activation without physiological calcium oscillation is considerably affected by the cytoplasmic maturity of the oocyte, including factors such as mitochondrial functionality, endoplasmic reticulum calcium stores, and maternal age [43]. Ionophore-induced activation elicits a singular, non-repetitive calcium transient that, although facilitates fertilisation, fails to replicate the pulsatile dynamics characteristic of natural fertilisation [44]. These dynamics are associated not only with oocyte activation but also with subsequent events, including zygotic genome activation (ZGA), epigenetic reprogramming, and the synchronisation of embryonic cleavage [45].

In practice, AOA is typically performed immediately after ICSI, during the brief interval post-injection when the oocyte remains responsive to activation signals. This is primarily intended for couples experiencing recurrent TFF, particularly when the male partner has a condition that adversely affects PLCζ expression, such as globozoospermia, macrozoospermia, or some forms of teratozoospermia [46,47]. In numerous instances, employing ICC on spermatozoa to detect the absence of PLCζ substantiates the focused application of AOA, which enhances fertilisation rates and the developmental potential of embryos [24,48].

AOA has been beneficial for unexplained fertilisation failure and female factor infertility, particularly in older women or those with DOR. It has also been beneficial for male factor infertility. In such instances, oocytes may exhibit diminished responsiveness to calcium due to age-related alterations in IP_3_ receptor density, depletion of endoplasmic reticulum calcium reserves, or mitochondrial dysfunction [32,49]. All of these factors can impede the oocyte’s ability to convert calcium influx into successful meiotic resumption.

Recent research indicates that AOA serves not only as a lifesaving measure but may also assist some demographics proactively. In several cohort studies, fertilisation rates significantly increased—from as low as 5–10% in prior ICSI cycles to over 60% with AOA—when addressing confirmed or suspected PLCζ-related activation problems [50]. In conjunction with these developments, there have been increased blastulation rates, improved transfer-quality embryos, and enhanced clinical pregnancy outcomes, particularly when molecular diagnostics are employed to determine treatment options [51].

From a molecular perspective, AOA functions not solely due to its generation of calcium signals, but also because the oocyte is capable of converting these signals into subsequent chemical responses. This entails activating the ERK1/2, CaMKII, and calcineurin pathways, which collectively govern cell cycle control, spindle integrity, and nuclear envelope reassembly [52]. Consequently, oocytes exhibiting impaired cytoplasmic maturation—an occurrence prevalent among older women desiring to conceive—may derive more advantages from AOA compared to their younger counterparts. The calcium influx may activate dormant signalling networks and preserve gametes that would not have otherwise matured [53].

Despite the favourable outcomes, AOA is not a universal solution. The induced calcium signal does not oscillate, which suffices for fertilisation but may not be optimal for long-term developmental programming, such as epigenetic remodelling [54]. Research on animals has raised concerns regarding the potential effects on chromatin structure and methylation patterns when calcium signalling is dysfunctional. To date, human data have not indicated any significant rises in birth abnormalities or developmental issues [55]. Nonetheless, it is crucial to select patients meticulously and monitor them over an extended period.

In addition to calcium ionophores, researchers have explored several physical and chemical methods to activate artificial oocytes, including piezoelectric stimulation. Piezo-assisted activation employs mechanical pulses generated by piezoelectric actuators to transiently permeabilize the oolemma, facilitating calcium influx independent of pharmacological agents. Calcium ionophores, such as ionomycin or A23187, function by chemically inducing a solitary calcium spike. Conversely, piezo activation can induce membrane depolarisation and intracellular signalling with minimal chemical exposure. Comparative studies demonstrate that calcium ionophores are more commonly utilised in clinical practice due to their user-friendliness and established safety in specific subpopulations, such as individuals with PLCζ deficiency or globozoospermia. Conversely, piezo stimulation may be beneficial in situations when oocytes have increased susceptibility to oxidative stress or when minimising medication exposure is desired. Nonetheless, piezo activation sometimes requires specialised apparatus and operator expertise, and its efficacy in human ART is less corroborated in comparison to calcium ionophores. Further comparative research is required to determine the superior option in many clinical scenarios, particularly for fertilisation rates, embryo quality, and epigenetic safety.

Another crucial consideration is the timing, dosage, and duration of exposure to ionophores. Improper utilisation of AOA, such as at excessive concentrations or prolonged exposure, may damage the cytoskeleton, induce abnormal pronuclear development, or activate parthenogenetic genes. It is imperative to adhere rigorously to established laboratory protocols to ensure safety and reproducibility.

In summary, artificial oocyte activation is a scientifically valid and therapeutically beneficial method to prevent fertilisation failure due to defective sperm-derived activation signals. AOA functions as PLCζ in its absence or malfunction, initiating the primary molecular steps that activate oocytes and facilitate the transition to early embryogenesis. The approach is most effective when used in conjunction with molecular diagnostics such as PLCζ immunolocalization. This enables ART therapy to be customised to each patient’s own pathophysiology. In the future, enhanced oscillation-mimetic pharmaceuticals and integrated stimulation techniques may render AOA more pertinent to the body and applicable in additional clinical contexts.

Understanding the distinctions between natural and artificial oocyte activation is crucial for enhancing assisted reproduction approaches, particularly in cases of fertilisation failure. Table 2 indicates that the sperm-specific isoform PLCζ is accountable for natural activation. It initiates a series of precisely regulated Ca^2+^ oscillations that persist for several hours. These oscillations are crucial for activating downstream molecular pathways, particularly CaMKII, PKC, and MAPK/ERK. These mechanisms together modify the cytoskeleton, alter the chromatin, and transition from maternal to zygotic stages. AOA utilising calcium ionophores such as A23187 or ionomycin induces a rapid influx of Ca^2+^ into the cytoplasm. This is frequently sufficient to activate CaMKII and reinitiate meiosis via the APC/C complex; however, it does not mimic the PLCζ signalling pattern, which is rhythmic and compartmentalised. This indicates that activities regulated by PKC, such as the exocytosis of cortical granules and cytoplasmic remodelling, may not occur simultaneously or may not occur at all, potentially impacting subsequent developmental stages. Ionophore-induced Ca^2+^ spikes can lead to mitochondrial Ca^2+^ overload, resulting in the production of ROS and imposing stress on the endoplasmic reticulum (ER). The oocyte’s buffering mechanisms typically compensate for this disturbance; however, such stress may be particularly detrimental for older women or those with suboptimal oocyte quality. Despite these issues, AOA remains an effective treatment for individuals with specific genetic deficits, such as PLCζ deficiency, globozoospermia, or OAD. When employed judiciously, particularly in conjunction with molecular diagnostics, AOA can enhance fertilisation, augment the quantity of embryos, and elevate clinical pregnancy rates in some populations.

## 4. Clinical Outcomes of AOA in Fertilisation Failure and Poor Responders

Artificial oocyte activation has emerged as a scientifically valid method to restore fertilisation competence in instances of OAD, often resulting from issues with sperm-derived PLCζ. Calcium ionophores administered externally mimic the intracellular calcium oscillations essential for reinitiating the oocyte’s developmental programme [56]. Calcium signals function as second messengers at the molecular level, facilitating the activation of signalling networks that regulate early embryogenesis, meiotic egress, and cytoplasmic maturation. The rationale for AOA is grounded not merely in observations, but in a profound comprehension of the disruptions in signal transduction cascades associated with aberrant fertilisation [52].

In typical circumstances, PLCζ is provided by sperm hydrolyses PIP2 to produce inositol IP_3_, which interacts with IP_3_R1 located on the ER. This induces the release of Ca^2+^ in pulses within the cell. Ca^2^^+^ oscillations persist for several hours post-fertilisation, initiating a cascade that activates the APC/C via the calmodulin–Ca^2^^+^–CaMKII pathway [57,58]. Upon activation of APC/C, ubiquitin is conjugated to cyclin B, leading to its degradation in the proteasome and the inhibition of the CDK1–cyclin B complex (MPF). This halts the oocyte at metaphase II. As MPF activity diminishes, the egg resumes meiosis, expels the second polar body, and forms the female pronucleus [59]. DAG synthesis simultaneously stimulates Ca^2+^-dependent PKC isoforms, which alter the structure of the actin cytoskeleton, facilitate the exocytosis of cortical granules, and prevent polyspermy by modifying the structure of the zona pellucida [60].

In cases of globozoospermia, certain forms of teratozoospermia, and unexplained fertilisation failure, the PLCζ signalling axis remains inactive. This maintains the egg cytoplasm in meiotic suspension [61]. Exogenous calcium ionophores such as ionomycin or A23187 circumvent this obstruction by directly permeabilizing the oolemma to Ca^2+^, initiating a global Ca^2+^ influx within the cell that artificially activates the CaMKII-APC/C pathway. This artificial activation does not replicate the frequency or length of natural oscillations; yet, it appears sufficient to initiate the molecular mechanisms required for successful fertilisation and early zygotic development [8].

Re-establishing this molecular axis significantly impacts clinical outcomes. Nicholson et al. conducted a pioneering study including 39 couples who had previously experienced complete or nearly total fertilisation failure, undergoing ICSI-AOA with calcium ionophore. Although the quantity of recovered oocytes was identical for both standard ICSI and AOA cycles, the fertilisation rate significantly increased from 7.1% to 57.2% following AOA. In a cohort with immunocytochemically confirmed PLC impairment, the fertilisation rate increased from 4.6% to 66.3%. The results indicate that the Ca^2+^ influx induced by ionophore therapy can substitute for the absence of sperm-derived activation, reinitiating the molecular processes essential for meiotic egress, pronuclear formation, and subsequent mitotic divisions [50].

The outcomes of this restored fertilisation cascade were seen at both the embryonic and clinical levels. AOA facilitated fertilisation and augmented the quantity of viable embryos, resulting in a rise in fresh embryo transfers from 33.3% to 94.6% [50]. The rates of clinical pregnancy and live birth significantly increased, underscoring the critical importance of artificially reinstating Ca^2+^-dependent signalling pathways [50]. These findings substantiate the notion that AOA is not merely a method to salvage unsuccessful laboratory fertilisation, but rather a legitimate therapy that addresses a specific molecular issue within the fertilisation signalling network.

Table 3 indicates that multiple clinical studies have demonstrated that AOA is beneficial in addressing PLCζ deficiency or diminished cytoplasmic competence, regardless of whether it is confirmed or presumed. Nicholson et al. examined 39 couples who had previously undergone TFF and discovered that the FR increased from 7.1% to 57.2% following AOA with CI. This was significantly more pronounced (up to 66.3%) in persons with immunocytochemically confirmed PLCζ− status, alongside improved ET availability and elevated CPR and LBR [50]. Kaur et al. examined POSEIDON-classified DOR women and discovered that AOA marginally increased FR (2.16 to 2.42) and significantly improved Grade A embryo production. The increase in CPR was not statistically significant (NS). These data suggest that AOA may aid in restoring calcium sensitivity in oocytes with metabolic dysfunctions [62]. Similarly, Tsai et al. discovered that AOA enhanced cleavage rates and Day 3 embryo quality in women aged 40 and above. Multivariate research established that AOA was an autonomous predictor of improved embryological outcomes, likely due to its restoration of impaired Ca^2^^+^–CaMKII–APC/C signalling [49].

However, not all studies support the efficacy of AOA in women with diminished ovarian reserve. In a rigorously structured prospective study, Aytaç et al. evaluated the impact of AOA in conjunction with calcium ionophore in women with diminished ovarian reserve undergoing ICSI, demonstrating no significant improvement in fertilisation rate, embryo quality, or clinical pregnancy rate relative to standard ICSI without AOA [63]. The statistics suggest that the benefits of AOA in this group may not be generally applicable, underscoring the need for tailored patient assessment. Variations among studies may stem from discrepancies in oocyte quality, the criteria employed to determine DOR, the techniques utilised for AOA, and the outcomes evaluated. Therefore, while AOA may benefit specific DOR patients, caution is required in generalising its effectiveness without further categorisation.

Tejera et al. demonstrated that AOA significantly impacted CCR, which decreased markedly from 69% to 22% in poor responders with prior suboptimal embryo development. This indicates that AOA is an efficient and economical method for rescuing oocytes, particularly in IVF patients experiencing repeated failures [1]. When applied appropriately, these data demonstrate that AOA can successfully bypass defective sperm-derived signals, enhance fertilisation, and promote embryo development and therapeutic results, particularly in PLCζ− or metabolically compromised oocytes.

Furthermore, AOA has been beneficial not just for PLCζ-mediated OAD but also for individuals with DOR and cytoplasmic incompetence, conditions frequently associated with calcium homeostasis disturbances or reduced ER Ca^2+^ reserves. Kaur et al. discovered that AOA led to a significant increase in fertilised oocytes (2.42 vs. 2.16), cleavage-stage embryos (2.32 vs. 1.96), and grade A embryos (1.52 vs. 1.04) in women who satisfied the POSEIDON criteria. The probable mechanism involves the restoration of Ca^2+^-sensing sensitivity in ageing oocytes or those with metabolic dysfunctions. These oocytes may exhibit diminished IP_3_R expression, disordered endoplasmic reticulum, or altered expression of CaMKII isoforms. Although the increases in implantation and pregnancy rates were not statistically significant, the molecular alterations observed in early developmental milestones warrant the application of AOA in patients whose oocytes may exhibit suboptimal responses to endogenous activation stimuli [62].

Tsai et al. discovered additional evidence for this in adults aged 40 and above. AOA treatment was associated with increased cleavage rates and a greater proportion of high-quality Day 3 embryos. This age-related reaction to AOA may be attributed to diminished ATP levels in the cytosol, impaired Ca^2+^ release from the ER, or alterations in calcium buffering proteins, all of which might hinder the resumption of meiosis. The research employed multivariate regression analysis to determine that AOA serves as an independent predictor of embryo quality. This indicates its potential to rectify molecular issues in calcium signalling pathways that arise with maternal ageing [49].

In a comprehensive ART context, Tejera et al. discovered that AOA significantly reduced the cycle cancellation rate from 69.3% to 22.7% in patients with a history of poor embryo development. This study indicates that addressing a single biological constraint—insufficient oocyte activation—can salvage entire treatment cycles that would otherwise be unsuccessful, hence reducing the stress and cost associated with repeated IVF failures [1].

The clinical efficacy of AOA is directly correlated with its molecular precision. It operates at a crucial signalling juncture that regulates the transition from metaphase arrest to embryonic development. By chemically reinstating this communication circuit, AOA enables a defective fertilisation process to persist [64]. Nonetheless, its efficacy significantly relies on the selection of appropriate patients, as the molecular profile of fertilisation failure varies across cases. Physicians can employ AOA in a focused, mechanism-oriented manner by integrating diagnostic methods such as PLCζ immunolocalization assays with morphokinetic profiling and the patient’s medical history. When employed alongside molecular diagnostics and appropriately timed during oocyte development, AOA is an effective intervention that can facilitate fertilisation, enhance embryo quantity, and improve the likelihood of a successful pregnancy in patients unresponsive to alternative therapies [49].

Despite AOA demonstrating favourable outcomes, certain patients may continue to experience difficulties conceiving, even with consistent calcium ionophore therapy. In these cases, alternative or modified techniques have been explored to tackle persistent oocyte activation deficiency. An alternative is to employ calcium ionophore for dual exposure or extended incubation, which prolongs the activation signal and may more accurately replicate elevated intracellular calcium levels. Studies demonstrate improved fertilisation rates with extended or repeated ionophore incubation periods, particularly following initial AOA failures. This approach may facilitate the activation of oocytes that have poor responsiveness to calcium or are marginally immature in the cytoplasm. However, these treatments must be meticulously evaluated against the risk of cytotoxicity and strictly adhered to in order to prevent overexposure. These results underscore the imperative for protocol flexibility in ART laboratories, especially in intricate situations where standard AOA fails to enable effective activation.

## 5. Embryo Morphokinetics and Development After AOA

AOA has demonstrated efficacy in addressing fertilisation issues; nonetheless, it raises significant concerns regarding its impact on subsequent embryonic development. It is intriguing to observe how artificially generated calcium signals influence embryo morphokinetics, namely the precise timing, synchronisation, and dynamics of early cleavage events. These characteristics are not merely observable, they also signify the molecular interactions occurring during oocyte reprogramming and embryonic genome activation. The concern is that the calcium influx induced by ionophores, distinct from the typical oscillating Ca^2+^ pattern, may alter the intracellular signalling environment and diminish the cell’s developmental capacity.

### 5.1. The Physiological Function of Ca^2+^ Oscillations in Oocyte Activation and Cell Cycle Reprogramming

In mammals, effective fertilisation of an egg requires a precisely coordinated intracellular signalling cascade initiated by periodic increases in cytoplasmic Ca^2+^ concentration. The oscillations commence upon the fusion of sperm and egg cells, primarily due to the sperm-specific isoform PLCζ, which hydrolyses PIP_2_ into IP_3_ and DAG [7]. The produced IP_3_ disseminates through the ooplasm and binds to IP_3_R1 on the endoplasmic reticulum, resulting in the release of Ca^2+^ from internal reserves in a wave-like manner [65]. PLCζ is distinct from other PLC isoforms as it is only located in the sperm head. It enters the egg cytoplasm following ICSI or natural fertilisation and remains enzymatically active even in the absence of membrane attachment [30].

The periodic Ca^2+^ oscillations, occurring over several hours, serve not merely as an on/off mechanism for oocyte activation. They are dynamic signals that ensure downstream effector activation occurs at the appropriate moment and with the correct intensity [4]. CaMKII is a crucial target that auto-phosphorylates and degrades EMI2 and cyclin B1 through the activation of the APC/C. This disruption halts MPF (the CDK1–cyclin B complex), a crucial regulator that maintains the oocyte in MII arrest. Meiosis must recommence with the extrusion of tPB2 and the development of the female pronucleus. This can occur solely after the cessation of MPF activation [66,67].

Simultaneously, CaMKII and PKC activate additional pathways, including MAPK/ERK signalling, which facilitates cytoskeletal reorganisation, chromatin remodelling, and proper assembly of the meiotic spindle [68]. Oscillating Ca^2+^ signals regulate mitochondrial localisation, which is crucial for optimising ATP production during energy-demanding activities such as CG exocytosis [69]. This event alters ZP glycoproteins and increases the rigidity of the zona matrix, hence preventing polyspermy, which is crucial for safeguarding against chromosomal aneuploidy.

The efficacy of Ca^2+^ oscillations is contingent upon their precise timing. The consistent, rhythmic pattern ensures that molecular switches activate incrementally, hence reducing the likelihood of asynchronous or premature meiotic exit. The frequency and amplitude of the oscillations are meticulously calibrated to ensure optimal activation of CaMKII and to prevent detrimental pathways such as apoptosis, which may occur if Ca^2+^ influx is excessively high or erratic [70].

These intracellular signals facilitate EGA, a critical juncture in development when transcriptional regulation transitions from maternal mRNA to the zygotic genome. Artificial activation techniques that alter Ca^2+^ dynamics could potentially modify chromatin accessibility, histone acetylation state, or DNA methylation patterns. This may impact the long-term viability of embryos and epigenetic transmission [71].

Finally, the mitochondrial–Ca^2+^ axis facilitates the uptake of Ca^2+^ into mitochondria, hence enhancing oxidative phosphorylation (OXPHOS). This not only sustains ATP generation but also maintains the equilibrium of reactive oxygen species within cells. Alterations in this delicate axis, whether induced by abnormal oscillatory kinetics or external activation, may impair the embryo’s developmental capacity [72,73].

PLCζ-induced Ca^2+^ oscillations serve as crucial molecular signals in fertilisation, meticulously coordinating cytoplasmic and nuclear processes in the egg to initiate embryogenesis [44]. They facilitate a seamless transition from a meiotically arrested oocyte to a totipotent zygote by integrating multiple downstream signalling pathways, including CaMKII, MAPK, APC/C, and mitochondrial bioenergetics. To maintain developmental authenticity, any alterations or counterfeit reproductions of this process, such as in AOA, must be meticulously calibrated to align with the natural pattern [74].

### 5.2. Calcium Ionophore and Its Non-Oscillatory Signalling Profile

Calcium ionophore-mediated AOA is a therapeutically advantageous yet biochemically uncomplicated substitute for the intricate, oscillatory Ca^2+^ signalling that occurs during natural fertilisation [75]. Ionomycin and A23187 are small, lipophilic chemicals that facilitate the translocation of divalent Ca^2+^ ions across the oolemma by creating hydrophilic holes or mobile ionophores inside the lipid bilayer [76]. These ionophores do not induce properly calibrated and temporally spaced PLCζ-mediated Ca^2+^ oscillations [77]. They induce a singular, monophasic increase in cytosolic Ca^2+^ concentration by facilitating the passive influx of exogenous Ca^2+^ into the ooplasm.

This abrupt and singular increase in Ca^2+^ bypasses the endogenous IP_3_–IP_3_R1–ER pathway, hence halting the essential regenerative feedback loops required for sustained oscillations. PLCζ continuously hydrolyses PIP_2_ to produce IP_3_, which activates IP_3_R1 in a cyclical manner, facilitating regulated Ca^2+^ release from endoplasmic reticulum reserves. CICR sustains these oscillations; however, they are meticulously regulated by inhibitory feedback mechanisms including Ca^2+^-sensitive phosphatases, calmodulin, and SERCA pumps, which restore Ca^2+^ to the endoplasmic reticulum to recalibrate the system. Calcium ionophores disrupt this self-regulating system, resulting in a global, unregulated, and transient surge of Ca^2+^ that lacks compartmentalization or temporal organisation [78].

The efficacy of ionophore-mediated activation at the molecular level is contingent upon the intensity of the Ca^2+^ spike, which must be sufficient to initiate CaMKII autophosphorylation and the degradation of EMI2. Activation of CaMKII results in the phosphorylation of CDC20, thus activating the APC/C complex. APC/C attaches ubiquitin to cyclin B1, leading to its degradation in the proteasome and cessation of MPF activity (CDK1–cyclin B1), hence allowing the oocyte to exit MII arrest [79]. Relying just on a singular transient Ca^2+^ influx may be insufficient to effectively activate or deactivate these molecular switches, particularly when the activation threshold for CaMKII is not consistently met in all oocytes or in the cytoplasmic microdomains where Ca^2+^ concentrations fluctuate [80].

The PKC pathway, typically activated by the simultaneous generation of DAG and Ca^2+^, is predominantly inactive during ionophore-based AOA due to the inability of ionophores to stimulate PLC enzymatic activity [81]. Consequently, essential PKC-mediated functions such as CG exocytosis and cytoskeletal remodelling may be compromised, potentially affecting the hardness of the ZP and its resistance to polyspermy. This raises concerns that oocytes triggered by ionophores may lack complete or timely cortical responses, particularly if they are aged or exhibit metabolic dysfunctions [82].

Ca^2+^ signalling is intricately associated with the mitochondrial compartment, which is crucial for fertilisation and the initial stages of embryonic development. Physiological Ca^2+^ oscillations enhance the MCU complex’s capacity to absorb mitochondrial Ca^2+^, thereby augmenting OXPHOS, increasing ATP synthesis, and altering mitochondrial dynamics, including the equilibrium between fission and fusion. Ionophores can induce a fast, non-oscillatory Ca^2+^ spike that may overwhelm mitochondria with Ca^2+^, transiently alter the membrane potential (Δψm), and generate excessive ROS. This stress may result in mitochondrial failure, poor spindle organisation, or abnormal chromosomal segregation in compromised oocytes, particularly in older women with infertility issues [44,83].

There is also an absence of spatial targeting for the rise of Ca^2+^ induced by ionophores. During natural fertilisation, Ca^2+^ waves initiate at the point of sperm entry into the egg and propagate across the oocyte. This facilitates the accurate identification of signalling events, such as the targeted activation of CaMKII adjacent to the metaphase spindle or the Ca^2+^-dependent alteration of actin filaments at the cortex [35]. In AOA, the ionophore induces a uniform increase of Ca^2+^ in the cytoplasm, which is not associated with any particular microdomains in the cortex or spindle [84]. This may indicate that critical phases in cytoplasmic maturation are overlooked or that actin–microtubule interactions are improperly synchronised.

The quality of Ca^2+^ signalling may influence the precision of EGA and chromatin remodelling regarding subsequent nuclear reactions. Histone modification dynamics are associated with oscillatory Ca^2+^ signalling, particularly via HDACs and HATs that respond to Ca^2+^ levels and kinase signalling [85]. A non-oscillating profile could alter the mechanisms by which transcription factors enter the nucleus or disrupt the disassembly and reassembly of the nuclear membrane. This may alter gene expression in the early zygote and the synchronisation of blastomeres [86].

Numerous clinical studies indicate that calcium ionophore-induced AOA is sufficient to facilitate fertilisation in patients with globozoospermia, PLCZ1 mutations, or idiopathic ICSI failure, despite considerable theoretical risks [87]. The variability in oocyte quality and intracellular Ca^2+^ handling indicates that the application of ionophore-based AOA may not consistently yield effective results. To predict AOA’s response, one must understand the oocyte’s intrinsic buffering systems, which encompass SERCA, PMCA, calbindin, and parvalbumin.

In summary, calcium ionophores effectively circumvent natural fertilisation signals, although at the expense of physiological integrity. Their non-oscillatory Ca^2+^ influx activates just a limited portion of the intrinsic signalling system, which is typically sufficient to induce MPF collapse and complete meiosis, yet is sometimes inadequate for comprehensive cytoplasmic reprogramming or enduring epigenetic stability. Future advancements in AOA may concentrate on methods that replicate oscillations, such as administering low-dose ionophore pulses repeatedly or utilising synthetic PLCζ, to more accurately emulate the physiological circumstances of Ca^2+^-mediated oocyte activation.

### 5.3. Time-Lapse Imaging (TLI) Evidence: Kinetic Comparisons

TLI has emerged as a crucial instrument in embryology, enabling scientists to see embryonic development in real time and with great resolution from the zygote to the blastocyst stage [88]. This continuous imaging technique eliminates the necessity for embryo exposure during manual evaluations and facilitates precise measurement of morphokinetic parameters, including the timing of pronuclear formation and dissolution, intervals between cleavage events, durations of specific cell cycles, and the degree of blastomere symmetry [89]. These figures are associated with developmental competence, implantation viability, and live birth rates. They serve as the foundation for algorithmic embryo selection systems such as KIDScore™ and Eeva^®^ [90].

The use of calcium ionophores for AOA necessitates the consideration of TLI to determine whether bypassing normal Ca^2+^ oscillations alters embryonic motility or increases susceptibility to developmental programming issues. Natural fertilisation induces a prolonged series of Ca^2+^ transients mediated by sperm-derived PLCζ [91]. Conversely, ionophore-mediated AOA induces a rapid, non-repetitive Ca^2+^ influx that is less complex in both spatial and temporal dimensions compared to endogenous signalling. The disparities raise valid concerns regarding their impact on subsequent processes critical for early cleavage events, including embryonic genome activation, cytoskeletal remodelling, and organelle coordination.

Numerous significant morphokinetic markers are influenced by alterations in Ca^2+^ signalling.

- tPB2 (time to second polar body ejection) indicates the deactivation of MPF, signifying the cell’s exit from meiosis [92].
- tPNa/tPNf (pronuclear appearance/fading) indicates that chromatin is becoming less compact, pronuclei are developing, and syngamy is commencing [93].
- The intervals t2–t8 (cleavage timings) and cc2/cc3 (cell cycle phases) indicate the progression of mitosis [94].
- The s2/s3 scores indicate the collaborative efficiency of the blastomeres over time [95].

Research conducted by Martínez et al. and Göde et al. indicates that embryos treated with AOA exhibit minor alterations in certain parameters, such as a postponed tPB2 and, in some instances, an accelerated t3. Nonetheless, there is no notable alteration in cleavage synchrony, symmetry, or rates of blastocyst development [96,97]. These alterations are probably attributable to modifications in the CaMKII–APC/C–MPF signalling mechanism. In delayed tPB2, the ionophore-induced Ca^2+^ spike may be insufficient to completely activate CaMKII in oocytes lacking adequate ER Ca^2+^ stores or lacking cytoplasmic maturity [98]. This may result in a more gradual breakdown of EMI2 and a slower collapse of MPF. Conversely, early t3 may indicate premature reactivation of CDK1 or a modification in the regulation of the mitotic checkpoint due to unsynchronised spindle formation.

TLI is unable to elucidate the composition of these divides, although their significance remains paramount. For instance, chromatin misalignment or spindle assembly issues may occur without altering the timing of cleavage. The SAC ensures that chromosomes are correctly attached to the spindle prior to the commencement of anaphase. This occurs through a sequence of phosphorylation events involving Aurora kinases, MAD2, and BUB1, all of which are responsive to ATP concentrations and Ca^2+^-regulated signalling [99]. If ionophore-induced Ca^2+^ influx temporarily halts ATP production in mitochondria or induces localised ROS, these checkpoints may be marginally compromised despite the continuation of cleavage processes [100].

Ca^2+^ signalling also influences the cytoskeleton. Ca^2+^-regulated actin-binding proteins, such as gelsolin, cofilin, and tropomyosin, govern the actin cytoskeleton, which is crucial for spindle migration, blastomere symmetry, and cytokinesis. Ionophores may disrupt the development of the polarised cortical actin cap by inducing unlocalized Ca^2+^ surges [101]. This may alter the orientation of the first cleavage plane or the location of the pronuclei. These alterations may not be reflected in standard TLI assessments; however, they could influence the embryo’s interaction with the uterus thereafter.

In the context of mitochondrial function, standard fertilisation induces oscillations of Ca^2+^, prompting the MCU complex to periodically absorb Ca^2+^. This enhances OXPHOS and generates the ATP required for mitosis, spindle rotation, and cytoskeletal remodelling [102]. Ionophore-mediated AOA, however, can induce a transient influx of Ca^2+^ into the mitochondria, potentially leading to transitory depolarisation, alterations in the dynamics of mitochondrial fission and fusion (regulated by OPA1, DRP1, and MFN1/2), and increased ROS production [103]. These redox alterations can compromise the integrity of both nuclear and mitochondrial DNA, but they may not manifest until post-implantation.

TLI investigations, such as those conducted by Mateizel et al., revealed that despite concerns, there was no substantial difference in blastocyst quality, utilisation rate, or Day 3 embryo grading between the ionophore-treated and control groups. This indicates that the oocyte possesses considerable molecular flexibility. Even when upstream signalling deviates from typical physiological patterns, the ooplasm possesses mechanisms to safeguard itself against detrimental effects, including redundant calcium effectors, antioxidant responses, and chaperone-mediated folding of essential mitotic proteins [104].

However, even little alterations in the timing of cleavage may be consequential. Embryos exhibiting shorter cc2 or cc3 intervals are more predisposed to aneuploidy or chromosomal mosaicism, particularly in older women at the time of conception [105]. AOA may exacerbate this risk among those already experiencing cytoplasmic issues. Although current research suggests that morphokinetics are largely consistent, there is a paucity of prospective evidence regarding whether AOA embryos alter implantation processes, the communication between the endometrium and other cells, or the outcomes of placentation.

Although TLI provides substantial evidence suggesting that the morphological trajectory of development remains consistent post-AOA, it is imperative to perform molecular profiling, including transcriptomic, epigenetic, and metabolomic studies, to fully comprehend the subsequent impacts. Integrating TLI with non-invasive embryo assessments, such as spent media proteomics or miRNA analysis, may enhance the selection of embryos subjected to AOA, hence reducing excessive dependence on kinetic criteria alone.

In summary, time-lapse morphokinetics indicate that AOA utilising calcium ionophores may induce minor alterations in cleavage dynamics; however, it generally does not influence the structure or timing of embryonic development. The results indicate that human oocytes have resilience and can react to artificial stimulation. Nonetheless, due to Ca^2+^’s multifaceted roles in regulating mitosis, transcription, metabolism, and chromatin architecture, it remains crucial to employ both morphokinetics and molecular diagnostics in tandem to enhance the application of AOA in clinical IVF procedures safely.

### 5.4. Intracellular Buffering and Cytoplasmic Resilience

The human oocyte can tolerate non-physiological calcium influxes during AOA due to its sophisticated intracellular buffering mechanisms and cytoplasmic robustness. Upon the natural fertilisation of an egg by sperm, PLCζ is released, catalysing the conversion of PIP2 into IP_3_. This prompts calcium to exit the ER via IP_3_R1. This initiates calcium oscillations that are spatially and temporally regulated [17]. These oscillations initiate activities essential for oocyte activation, including meiotic resumption, second polar body extrusion, and cortical granule exocytosis. Conversely, AOA with calcium ionophores like A23187 bypasses the conventional pathway, resulting in an abrupt, non-oscillatory influx of calcium [37]. Despite the alteration, the oocyte possesses a sophisticated array of compensating mechanisms that prevent calcium overload and cellular apoptosis.

The endoplasmic reticulum is the primary reservoir for calcium within cells, and it actively reabsorbs cytosolic calcium via ATP-dependent sarco/endoplasmic reticulum calcium-ATPases (SERCA pumps) [106]. Calreticulin and GRP78/BiP are luminal chaperones that regulate this process and maintain calcium homeostasis. STIM1 serves as a calcium sensor within the endoplasmic reticulum. Should the endoplasmic reticulum deplete calcium or accumulate an excess of misfolded proteins due to a sudden influx of ions, the unfolded protein response (UPR) may be activated through the signalling pathways of IRE1, PERK, and ATF6 [107]. This adaptive response enhances the activity of molecular chaperones, facilitates the degradation of misfolded proteins, and, if stress persists, activates apoptotic pathways. In the majority of developmentally competent oocytes, these protective mechanisms prevent cell death, hence facilitating proper meiotic and mitotic progression [108].

Mitochondria function simultaneously as calcium buffers and metabolic integrators. They absorb additional calcium via the MCU complex, which accelerates the TCA cycle and generates more ATP, essential for energy-demanding processes such as spindle movement and protein refolding [109]. Excessive mitochondrial calcium intake, surpassing the buffering capacity, can lead to membrane depolarisation, the activation of the mPTP, and the release of apoptotic signals such as cytochrome c [110]. MICU1 and NCLX regulate calcium transport into and out of mitochondria, whereas fission and fusion proteins such as DRP1, MFN1, and OPA1 modulate mitochondrial dynamics. These pathways maintain mitochondrial health and preserve redox equilibrium [111].

Intracellular calcium levels significantly influence the actin cytoskeleton. Proteins such as gelsolin, cofilin, and formin-2 react to calcium signals by regulating the remodelling of filamentous actin. This is crucial for the extrusion of polar bodies, the migration of pronuclei, and the division of symmetric blastomeres [101]. Artificial calcium influx can disrupt actin organisation; however, Rho-family GTPases and calmodulin-mediated pathways maintain cortical stability. This elucidates the absence of significant alterations in cleavage symmetry or fragmentation in embryos derived from AOA-treated oocytes, as demonstrated in time-lapse morphokinetic tests.

During fertilisation, RNA-binding proteins such as CPEB1, DAZL, and PUMILIO regulate the selective activation of maternal mRNAs stored in the oocyte by cytoplasmic polyadenylation [112]. Calcium/calmodulin-dependent kinases, such as CaMKII, regulate these proteins. They can remain active even in the absence of fluctuating calcium levels, facilitating the prompt synthesis of crucial regulators such as MOS, ZAR1, and cyclin A2 [113]. Despite the absence of physiological calcium oscillations, translational activation appears to be predominantly maintained during AOA cycles, facilitating normal early embryonic development.

Oxidative stress is an additional issue that arises with artificial calcium infusion. Mitochondrial calcium intake can enhance the production of ROS; however, the oocyte possesses robust defences against these detrimental agents. SOD2, GPx, catalase, and peroxiredoxins are enzymes that work in conjunction with non-enzymatic antioxidants such as GSH to mitigate ROS and safeguard against lipid peroxidation, protein oxidation, and DNA damage [114]. The Nrf2 pathway enhances these systems by elevating antioxidant response elements when redox equilibrium is disrupted. This enhances the stability of the cell’s interior.

Ultimately, despite the occurrence of artificial activation, the integrity of the epigenome remains intact. Histone acetyltransferases and HDACs are calcium-sensitive enzymes that modify histones. DNMTs and TET enzymes affect DNA methylation patterns. These alterations remain largely consistent in oocytes subjected to AOA treatment. The regulated mobility of the nuclear envelope, governed by CaMKII and CDK1 via Lamin A/C phosphorylation, facilitates precise pronuclear assembly and chromatin remodelling. The successful occurrence of embryonic genome activation indicates that ionophore treatment does not substantially influence these epigenetic mechanisms.

In summary, the cytoplasmic resilience of the oocyte arises from a complex interplay of calcium buffering, organelle coordination, cytoskeletal remodelling, antioxidant defence, translational regulation, and epigenetic modulation. This network assists the oocyte in managing the influx of artificial calcium during AOA, hence maintaining its growth potential. Divergences in buffering capacities—attributable to maternal age, metabolic condition, or oocyte quality—underscore the necessity of evaluating each case individually when considering AOA in assisted reproductive techniques.

### 5.5. Considerations for Embryo Selection Algorithms

The increasing adoption of time-lapse imaging platforms such as EmbryoScope™, Geri™, and Eeva™ in assisted reproductive technology laboratories has transformed embryo assessment by facilitating continuous observation of critical developmental milestones, including tPNf (pronuclear fading), t2–t8 (cleavage stages), cc2/s2 (cell cycle synchronisation), and tB/tSB (blastulation and initiation of blastulation) [115]. Predictive models such as KIDScore™, iDAScore™, and Eeva™ utilise these kinetic factors to evaluate embryos. These models examine the temporal development of embryos and their likelihood of successful implantation and subsequent birth. However, the significance of these characteristics must be re-evaluated in the context of AOA, particularly when ionophore-induced Ca^2+^ surges trigger activation [116].

AOA induces a singular, abrupt surge in intracellular Ca^2+^, whereas PLCζ elicits physiological oscillations. This influences the initial molecular checkpoints that regulate meiotic initiation and embryonic genome activation. The timing of CaMKII activation, primarily reliant on Ca^2+^, may vary, hence altering the rate of EMI2 degradation and APC/C activation [66]. Consequently, tPB2 extrusion and tPNf may not align with conventional TL-based “optimal” windows, potentially resulting in otherwise healthy embryos being classified as delayed [117]. This desynchrony may induce classification bias, as TL techniques rely on datasets derived from non-AOA cycles.

Mitochondrial metabolic responses in AOA-treated embryos may vary due to alterations in mitochondrial Ca^2+^ uptake mechanisms. These processes are regulated by Ca^2+^-sensitive dehydrogenases such as PDH, IDH, and αKGDH. Alterations in ΔΨm, ATP availability, and ROS generation influence the timing of blastomere cleavage, spindle dynamics, and the fidelity of cytokinesis [118]. All of these alterations are indirectly quantified by the cc2, s2, and t3–t5 metrics. This complicates the comprehension of early cleavage processes, particularly when mitochondrial dysfunction is common in older women or those with immature oocyte cytoplasm [119].

Concurrently, ATP-dependent chromatin remodelling complexes (such as SWI/SNF and NuRD), HDACs, and TET enzymes facilitate nuclear remodelling and epigenetic programming. These events occur subsequent to Ca^2+^ signalling and may be influenced by alterations in timing under AOA. These pathways regulate chromatin accessibility and the initiation of transcription during ZGA [120]. They could alter the morphology of the nucleus, the reassembly of the nucleolus, and the velocity of transcriptome processes. These alterations are not immediately apparent in TL, but they may manifest as delays in the tPNf or t2–t3 intervals.

Significantly, TL grading systems increasingly employ AI-driven image analysis methods to assess blastomere symmetry, fragmentation, compaction quality, and blastocyst expansion. Alterations to the cytoskeleton induced by AOA can transiently disturb the symmetry of cleavage or the alignment of blastomeres, yet do not impair their growth capacity. These alterations are induced by calcium-dependent effectors such as gelsolin, cofilin, and mDia. Although these little structural variations are innocuous, they may be interpreted as indicators of a poor prognosis by an algorithm due to insufficient molecular context.

Research on gene expression in embryos from AOA cycles indicates the continued presence of pluripotency markers (OCT4, SOX2), trophectoderm lineage determinants (CDX2, GATA3), and maternal-to-zygotic transition genes (ZSCAN4, DPPA2/4) [121]. These findings corroborate the notion that molecular programming remains consistent, despite alterations in morphokinetics. Selection algorithms that solely consider morphokinetics may discard viable embryos due to timing discrepancies that are either insignificant or misinterpreted [122].

Incorporating cycle metadata into TL-based embryo evaluation would constitute a significant enhancement. This would encompass details such as the completion of AOA, the utilisation of ionophore, PLCζ status, and maternal variables including AMH levels or oocyte maturity scores. Incorporating molecular indications such as pCaMKII, ΔΨm, mtDNA copy number, or cytoplasmic redox indices (GSH:GSSG ratio) may enhance the ability to predict embryo viability beyond mere assessment of morphology or motility.

Ultimately, TL should be employed in AOA-treated embryos to advance towards “molecularly aware” AI frameworks. Training algorithms on AOA-specific datasets is crucial to ensure their capability to manage spontaneous variations in kinetic events that do not influence embryonic epigenetic reprogramming or lineage commitment. The subsequent advancement in tailored, mechanism-based ART involves transitioning from phenotype-only embryo profiling to multi-parametric embryo profiling.

### 5.6. Future Perspectives: Advancing Oscillation-Copying AOA

Contemporary AOA methodologies employing Ca^2+^ ionophores like A23187 and ionomycin induce abrupt, non-oscillatory [Ca^2+^]_i_ spikes that bypass the conventional PLCζ–PIP2–IP_3_–IP_3_R1 pathway. These singular surges suffice to dismantle EMI2 and deactivate MPF via CaMKII-γ-dependent APC/C–CDC20 activation; however, they fail to replicate the requisite natural oscillation pattern essential for the sequential activation of maternal effectors. Ionophore-induced signals lack the temporal encoding fidelity of natural fertilisation, hence failing to facilitate precise CaMKII phosphorylation–dephosphorylation cycles, regulate the MOS/ERK axis, or sustain translational activation via CPEB1 polyadenylation. This may diminish the efficacy of post-zygotic reprogramming, chromatin accessibility, and mitotic synchronisation.

Micro-injection of hPLCζ-mRNA is one method employed by scientists to generate oscillatory patterns. This technique delivers translationally active synthetic mRNA straight into the MII cytoplasm. This reinstates the release of ER-Ca^2+^ regulated by IP_3_R1 every 10 to 15 min, initiating synchronised CaMKII bursts, EMI2 degradation, CDK1–Cyclin B1 disassembly, and the progression from metaphase to anaphase. In murine and non-human primate oocytes, hPLCζ induces normal tPB2, tPNf, and t2 kinetics while maintaining ΔΨm, ATP production (by CS, SDH, and COX1), and the transcriptional activation of genes related to ZGA (including ZSCAN4, KLF17, and DPPA5). Furthermore, oocytes activated by hPLCζ-mRNA exhibit appropriate biallelic methylation patterns at imprinting control regions (such as H19-DMR and PEG3-DMR), maintain elevated H3K27me3 levels in maternal loci, and reduce the levels of apoptotic markers (including BAX and AIFM1).

Researchers have attempted to utilise microfluidic perfusion or time-regulated piezo-actuation to administer Ca^2+^ ionophores in pulses to replicate oscillations. Repetitive, submaximal [Ca^2+^]_i_ peaks more accurately synchronise CaMKII–ERK–p90RSK signalling cycles, resulting in the progressive degradation of mRNAs featuring CPEs and the enhancement of maternal RNA translation, including CCNB2, PTTG1, and CDC6. This technique enhances spindle anchoring by stabilising TPX2–Aurora A–γ-tubulin complexes, which are crucial for the formation of symmetric blastomeres. It also enhances actin cytoskeleton remodelling via mDia1, cortactin, and p-cofilin.

ChR2, CatCh, and Opto-ORAI exemplify optogenetic methodologies that enable precise modulation of Ca^2+^ influx through light stimulation. These systems offer optimal temporal control. They activate STIM1–ORAI1 CRAC channels to release ER-Ca^2+^ in PLCζ-mimetic bursts. Utilising genetically encoded calcium indicators (GECIs) like GCaMP6, jRCaMP1b, or CEPIA, optogenetic AOA enables real-time modulation of oscillation frequency and duration. Light-modulated Ca^2+^ entrainment in engineered hESC oocytes activates NFATc1 nuclear translocation, CREB phosphorylation, and downstream ZGA markers (including DPPA3, KDM4D, and SMARCA4), while preserving chromatin structure and the formation of nucleolar bodies.

Recent designs are exploring synthetic Ca^2+^ nanotransducers, such as UCNPs–Ca^2+^ chelates, which emit ions upon exposure to NIR light. These would provide non-invasive, programmed alterations in intracellular calcium levels. It is possible to link CaM–CaMKII responsive peptides or ER-targeted buffering domains, such as calreticulin-fused EGTA, to these nanodevices to create localised Ca^2+^ microdomains according to your specifications.

Translational integration necessitates rigorous evaluation of developmental capacity post-AOA. Multi-omic profiling, encompassing single-cell RNA sequencing, ATAC sequencing, and reduced representation bisulfite sequencing, has revealed that AOA simulating oscillation reinstates traditional ZGA waves, enhances maternal RNA clearance transcripts (notably BTG4 and CCR4-NOT), and sustains TET3-mediated 5hmC oxidation. Chromatin immunoprecipitation indicates that H3K4me3, H3K36me2, and H2A.Z are appropriately localised, whereas mitochondrial proteomics reveals consistent expression of ATP5A1, TOMM20, and ND5. Immunocytochemistry is significant as it demonstrates that spindle checkpoint proteins, such as BUB1 and MAD2L1, consistently localise in the same region, indicating the continued functionality of the spindle assembly checkpoint response.

Regulatory and bioethical concerns are paramount. To prevent hPLCζ-mRNA from eliciting an excessive immune response, it must be optimised (for instance, by substituting certain nucleotides with pseudouridine or N1-methyl-pseudouridine), capped with CleanCap™ or ARCA, and polyadenylated through enzymatic tailing to inhibit premature degradation. By engineering UTRs and incorporating miRNA-122 target sites for rapid degradation in non-oocyte contexts, the risks of off-target expression can be mitigated. Delivery methods such as ICSI co-injection, electroporation, and microfluidic–liposome hybrid platforms are being enhanced in accordance with GMP regulations.

Future TL-based embryo evaluation algorithms must be modified to comprehend developmental changes during oscillatory AOA. Integrating kinetic metadata (such as the tPB2–tPNf delay index, s2 width, and ΔΨm fluctuation rate) with non-invasive spent media metabolomics (including the lactate/glucose ratio, amino acid turnover, and ROS markers) alongside AI-enhanced image analysis will enable the selection of embryos that align with the distinct dynamics of oscillatory signalling.

The forthcoming generation of AOA transcends static calcium release and endeavours to replicate the entire spatiotemporal signalling framework of fertilisation. The integration of transcriptomics, optogenetics, synthetic biology, and intelligent embryology is transforming AOA from a mere rescue technique into a configurable, biomimetic fertilisation system that enhances cellular fidelity, developmental competence, and therapeutic outcomes.

## 6. Cytoplasmic Buffering and Adaptive Processes

Following AOA-induced elevation of [Ca^2+^]_i_ via ionophore or hPLCζ-mRNA-mediated IP_3_ production, the MII oocyte activates a complex buffering network to maintain ionic equilibrium and facilitate meiotic progression. IP_3_R1 is situated in the endoplasmic reticulum and facilitates CICR. The process is rapidly halted by SERCA2b-mediated Ca^2+^ reuptake into the ER lumen, regulated by CRT and CNX during luminal Ca^2+^ saturation. The transport of Ca^2+^ between the endoplasmic reticulum and mitochondria is facilitated when IP_3_R1, Grp75, and VDAC1 are interconnected inside MAMs and stabilised by MFN2 and PACS2. This enables the precise delivery of [Ca^2+^] to the mitochondrial matrix [123].

The MCU–MICU1/2 axis facilitates the influx of Ca^2+^ into mitochondria, hence enhancing the activity of PDH, IDH3, and αKGDH. This enhances TCA cycle flux and promotes NADH/FADH_2_ production for electron transport chain complexes I–IV [124]. Elevated Ca^2+^ levels result in electron leakage from Complex I, leading to the formation of ROS, which subsequently triggers the dissociation of KEAP1–NRF2, allowing it to translocate to the nucleus [125]. NRF2 associates with AREs in the promoters of antioxidant genes such as SOD2, PRDX3, GPX1, and HO-1 to prevent the accumulation of ROS. Excessive ROS activate ATM/ATR kinases, which phosphorylate H2AX (γH2AX) and recruit BRCA1/53BP1 repair foci to save DNA [126].

The AMPK–LKB1 complex identifies diminished ATP:AMP ratios and activates the ULK1–BECN1–ATG13–FIP200 autophagy initiation complex. PINK1–PRKN translocates to the outer mitochondrial membrane of depolarised mitochondria (low ΔΨm), initiating mitophagy by the ubiquitination of MFN1/2 and VDAC1 [127]. The CREB1–Ca^2+^ regulation facilitates mtDNA replication and the proliferation of new mitochondria through the TFAM–POLG–PGC1α axis.

In the cytosol, Ca^2+^ buffering proteins (CALB1, PV, S100B, SRI) exhibit a considerable affinity for excess Ca^2+^, hence altering the surrounding microdomains. The phosphorylation of CaMKII-cofilin, the severing activity of GSN, and Arp2/3-dependent nucleation collectively regulate the remodelling of F-actin [127]. To facilitate spindle movement, actin must polymerise with the assistance of PFN1 and be capped by CAPZ–Tmod3. This pertains to GTPase RAC1–CDC42–PAK1 signalling.

Phosphorylation of CPEB1 by CaMKII initiates translation reprogramming by recruiting CPSF and GLD2 to extend the poly(A) tails of maternal mRNAs such as CCNB1, CDK1, CDC25B, and BUB1B. The mTORC1–RPS6KB1–EIF4EBP1 pathway enhances translational efficiency, facilitating the assembly of the EIF4E–EIF4G–PABP complex to initiate translation with a cap structure. REDD1, Sestrin2, and TSC2 regulate metabolic checkpoints concurrently with translational activation.

Ca^2+^-responsive chromatin remodellers maintain epigenetic stability following AOA. The CaM–CaMKII-mediated nuclear export of HDAC4 alleviates the suppression of H3K9ac/H4K12ac marks, thereby activating HAT1 and EP300. The NuRD (CHD4/MTA2), INO80, and BAF (SMARCA4/BAF155) complexes facilitate the repositioning of nucleosomes to enable ZGA functionality. The deposition of H3.3 mediated by HIRA–ASF1A/B and the chaperoning by NPM2 re-establish chromatin accessibility. Simultaneously, DNMT3A/L and TET3 facilitate the reprogramming and oxidation of cytosine methylation (5mC → 5hmC → 5fC).

RCC1 establishes a Ran–GTP gradient that facilitates the assembly of ELYS–NUP107–SEH1 complexes to reconstruct nuclear pore complexes (NPCs). The control of LMNB1–PP2A–CDK1 stabilises the nuclear envelope, ensuring that the formation of the nuclear pore occurs with appropriate dimensions, chromatin condensation, and importin α/β transport.

Prolonged alterations in [Ca^2+^]_i_ initiate the unfolded protein response in the endoplasmic reticulum via the IRE1α–XBP1, PERK–ATF4, and ATF6–S1P/S2P pathways. These mechanisms enhance the endoplasmic reticulum’s capacity for protein folding by elevating the concentrations of BiP/HSPA5, ERdj5, and GRP94. ATF5–CHOP signalling during concurrent UPR^mt^ induces the synthesis of HSP60, LONP1, CLPX, and mtHSP70, hence maintaining the assembly of the OXPHOS complex.

MII oocytes exhibit significant adaptive plasticity following artificial Ca^2+^ influx. This encompasses coordinated ionic buffering, metabolic adaptation, redox defence, translational activation, chromatin remodelling, nuclear reconfiguration, and proteostasis. These systems, which interact with many organelles and respond to Ca^2+^, ensure that development persists even when activated abnormally. Their preservation may be crucial for embryonic competence and enduring epigenetic stability following AOA.

## 7. Safety and Neonatal Outcomes of AOA

Typically, AOA is conducted with A23187 or ION. It induces non-oscillatory [Ca^2+^]_i_ transients that bypass hPLCζ-IP_3_R1 signalling. Despite being intentional, blastomeres from AOA exhibit preserved activation of MZT regulators, including BTG4–CNOT7-mediated maternal mRNA degradation, PAN2–PAN3 de-adenylation, and the upregulation of ZSCAN4, NELFA, and DUX4. The transcriptional waves of EGA remain unchanged, facilitated by the formation of RNA Pol II Ser5P foci and the recruitment of TFIIB/TFIID.

At the chromatin level, the export of HDAC4 is regulated by CaMKII, facilitating the initiation of transcription in the H3K9ac and H4K16ac states [128]. The activity of PRC2 (EZH2–SUZ12–EED) remains at bivalent promoters, preventing the activation of differentiation genes while permitting the expression of pluripotency-associated loci, such as NANOG, SOX2, and DPPA4 [129]. No reduction of H3K27me3 occurs in Hox clusters or imprinted loci. Preserving UHRF1–DNMT1 at replication foci and enhancing the DNMT3L–TET1 ratio sustains DNA methylation equilibrium.

In XX zygotes, AOA embryos exhibit steady XCI dynamics, characterised by XIST overexpression, macroH2A1 recruitment, and RPS4X silencing, indicating the efficacy of dosage compensation [130]. The demethylation of H3K27me3 by UTX and JMJD3 at active promoters facilitates lineage plasticity, while BRG1–BAF155 chromatin remodelling enhances accessibility to developmental enhancers.

The mitochondria in AOA embryos function normally at both structural and bioenergetic levels. The expression of MFN1/2, OPA1, and DRP1 maintains equilibrium between fission and fusion cycles. TMRE fluorescence verifies the preservation of ΔΨm, while intact ETC supercomplexes (CI + CIII + CIV) sustain ATP production through OXPHOS (CI–CV) [131]. Stable replication of mtDNA-CN was demonstrated using qPCR targeting ND1 and CYTB, whereas the expression of TFAM, TFB2M, and POLRMT validated the transcriptional competence of mtDNA.

The nuclear translocation of NRF2 facilitates the preservation of oxidative homeostasis by initiating the transcription of antioxidant response elements (AREs) such as PRDX1, SOD2, and GCLC. The activation of the PINK1–PRKN pathway remains within normal parameters, indicating an absence of excessive mitophagic turnover, while the LC3-II/I ratios demonstrate a consistent autophagic flux [132,133].

The proteostatic maintenance in AOA embryos is validated by the persistent signalling of UPR^ER^ via the ATF6, IRE1α-XBP1s, and PERK–eIF2α–ATF4 pathways. This facilitates the synthesis of HSPA5, HSP90B1, and PDIA6 without exhibiting indications of persistent ER stress, including CHOP overexpression. ATF5 and CHOP collaborate to maintain UPR^mt^ integrity by initiating the transcription of HSP60, LONP1, and CLPX [134].

The expression of FLT1, PGF, ENG, and VEGFA in the placenta post-implantation indicates successful trophoblast vascular remodelling. The conversion of spiral arteries remains unchanged, and the syncytiotrophoblast markers (GCM1 and ERVW-1) indicate that differentiation is preserved [135].

The γH2AX and comet assays indicate a paucity of double-strand breaks in the DNA. The apoptotic indicators (cleaved CASP3 and the BAX/BCL2 ratio) remain unchanged in the control embryos. Cell cycle markers such as p-RB, CDK2, and CCNE1 indicate that the G1–S transition is regulated, whereas the timing of mitosis is reflected by the kinetics of phospho-AURKA and CDC25B [136].

No methylation abnormalities are present at ICRs (including H19-DMR, KCNQ1OT1-DMR, and SNRPN-DMR) in postnatal molecular analyses of umbilical blood and buccal epithelium. MS-PCR and SNP microarrays do not detect any mosaic imprinting loss or uniparental disomy. No increase in aneuploidies or deleterious copy number variations occurs when karyotyping and whole genome sequencing are performed [137].

The neurodevelopmental assessment of AOA children indicates that their brain organisation and synaptogenesis remain intact. In CC, CST, and SLF, MRI-derived DTI exhibited normal fractional anisotropy and mean diffusivity. The theta/beta ratio and ERP latency from the qEEG indicate a normal electrophysiological condition. The Bayley-III, MSEL, and Vineland-3 behavioural assessments indicate that the child is performing satisfactorily across all developmental domains for their age. Epigenetic clocks applied to neonatal methylomes do not indicate an acceleration of biological ageing. The worldwide distribution of 5hmC and TET activity is normal. We maintain consistent telomerase activity (hTERT expression, TERC integrity) and telomere length (T/S ratio).

All data indicate that AOA-induced activation does not adversely affect early embryogenesis, epigenomic stability, cellular energy expenditure, or postnatal neurodevelopment, despite bypassing natural Ca^2+^ oscillatory rhythms. To ensure the safety of AOA in ART situations, we must implement continuous multi-omic tracking, high-resolution methylome–transcriptome coupling, and intergenerational surveillance.

## 8. Clinical Application and Patient Selection

When physiological PLCζ-induced Ca^2+^ oscillations fail to initiate MII departure, AOA has emerged as a compensatory strategy in ART. PLCζ initiates the hydrolysis of PIP_2_ during standard fertilisation, resulting in the release of IP_3_ that interacts with IP_3_R1 on the endoplasmic reticulum. This induces Ca^2+^ transients that activate CaMKII downstream. CaMKII phosphorylates EMI2, initiating SCF-mediated degradation. This liberates APC/C from its inhibition, allowing it to attach to CDC20 and ubiquitinate CCNB1. The reduction in CDK1 activity inhibits MPF function, hence facilitating tPB2 extrusion, cytoskeletal rearrangement, and PN formation. Concurrent activation of MAPK (ERK1/2) stabilises the spindle microtubules. Ca^2+^ facilitate cortical granule exocytosis, ensuring monospermic fertilisation through the cleavage of ZP2 and the cortical reaction.

If PLCζ is defective or absent, MPF remains stable, meiosis ceases, and OAD occurs. The standard WHO sperm criteria are still unable to identify this biochemical phenotype. Immunofluorescence utilising anti-PLCζ Abs provides a semi-quantitative assessment of cytoplasmic versus equatorial signals, whereas WB indicates the presence of full-length PLCζ isoforms. RT-qPCR and RNA-seq identify a reduction in PLCZ1 transcript levels or exon skipping events in translational diagnostics. Next-generation sequencing targeting PLCZ1 reveals pathogenic SNPs (such as p.H398P and p.I489F), and an in silico analysis of anticipated protein folding by homology modelling suggests structural instability or the absence of the catalytic domain.

Ca^2+^ imaging in Xenopus or murine oocytes subsequent to human sperm injection reveals the absence of intracellular calcium oscillations ([Ca^2+^]i). Fluo-4 AM or Fura-2-based ratiometric imaging reveals either monophasic transients or total Ca^2+^ quiescence, hence validating a PLCζ− profile. Additional evidence is derived from delayed tPNf, extended cc1, or asynchronous t2–t3 during TLI monitoring, suggesting that the cytoplasm is not completely active. Individuals with POR (e.g., AFC < 5, AMH < 1.1 ng/mL) experience exacerbated challenges in Ca^2+^ mobilisation due to mitochondrial malfunction, endoplasmic reticulum fragmentation, and diminished ATPase activity, which contribute to inadequate IP_3_R1 clustering and reduced endoplasmic reticulum reserves.

Patients selected for AOA comprise individuals who have experienced TFF following ICSI, GZSP with round-headed sperm morphology and acrosome loss, and those with unexplained fertilisation rates of less than 30% 2PN despite optimal TESE or ejaculated sperm exhibiting normozoospermia. In many instances, unsuccessful blastulation and low utilisation rates (ET + VIT/oocyte) indicate OAD. In women over 40, age-related reductions in MCU expression, elevated mitochondrial ROS, and dysfunctional SERCAs hinder the buffering and responsiveness of Ca^2+^, even in the presence of PLCζ+ sperm. This substantiates the role of AOA as a cytoplasmic enhancer.

The AOA technique typically employs A23187 (10 μM, 15 min) or ION (5 μM, 10 min), and is administered 30 to 60 min post-ICSI. The timing is crucial: administering it before to the redistribution of cortical granules or the formation of PN may result in anomalous cleavage. Rhod-2 AM or Indo-1 can be employed to quantify an increase in intracellular Ca^2+^, indicating that the cytoplasm is responsive. We utilise TLI to monitor the kinetic patterns of post-AOA embryos (e.g., s2 < 0.76 h, cc3 < 13 h) associated with high-quality blastocysts. Employing JC-1 to assess Δψm and MitoSOX to evaluate mitochondrial ROS enables the examination of mitochondrial competency post-activation.

Sperm parameters (PLCZ1 genotype, DNA fragmentation index, protamine/histone ratio), oocyte viability (cortical granule maturation, ER–Golgi interface integrity), and laboratory conditions (pH, O_2_ tension) all influence the efficacy of AOA. Patients with PLCζ− see a rise in fertilisation rates from around 5% to over 60% with AOA, along with elevated usable blastocyst rates. In PLCζ+ situations, however, AOA is minimally beneficial and may induce unnecessary alterations. Live-cell imaging of the delay in CaMKII activation, the lag in APC/C–securin turnover, and the duration of ERK nuclear exclusion may assist in identifying genuine AOA candidates.

In conclusion, the effective integration of molecular diagnostics (PLCζ, Ca^2+^ imaging, gene expression profiling) with embryologic dynamics (TLI morphokinetics, blastulation efficiency) constitutes the paramount aspect of tailored AOA in ART. As our understanding of the Ca^2+^–MPF–APC/C–EGA axis deepens, AOA will enhance in precision and use. It will transition from an empirical rescue methodology to a therapeutic instrument informed by genomes and proteomics.

## 9. Integration with Molecular Diagnostics and Novel Biomarkers

To transform AOA approaches into a precision-guided approach, it is essential to comprehend the molecular mechanisms underlying fertilisation failure, which is informed by MDx. PLCζ is the sperm-specific isoform of phosphoinositide-specific phospholipase C that initiates IP_3_-mediated calcium oscillations following ICSI [57]. It is at the core of this diagnostic framework. WHO-standardised semen analyses can provide extensive information regarding sperm count, motility, and morphology; nevertheless, they are unable of detecting proteomic anomalies. Patients exhibiting morphologically normal spermatozoa may yet possess PLCζ− profiles, indicative of an underlying molecular infertility phenotype [138]. It is essential to employ protein-level and gene-level assays to assess PLCZ1 expression to distinguish authentic OAD cases that qualify for AOA.

Utilising mAb against PLCζ facilitates the qualitative and semi-quantitative assessment of the localisation of sperm-specific proteins. Normo-functional sperm typically exhibit intense fluorescence at the postacrosomal sheath, whereas PLCζ− samples display either diffuse cytoplasmic staining or an absence of signal [139]. Confirmatory Western blot analysis utilising anti-PLCζ antibodies demonstrates the presence of full-length (~75 kDa) and truncated versions (~50–60 kDa) of PLCZ1. These may result from nonsense or splice-site mutations in PLCZ1 [140]. Numerous truncated isoforms lack the XY linker and EF-hand domains, which are crucial for binding to PIP_2_ and synthesising IP_3_. RT-qPCR may quantify PLCZ1 mRNA levels in concurrent transcript-level assessments, frequently indicating that defective samples exhibit mRNA levels exceeding a twofold reduction. RNA-seq provides exon-level resolution in translational contexts, enabling the identification of splice variants or allele-specific expression loss. This molecular information is essential in identifying mosaic GZSP or concealed mutational occurrences.

NGS mutation screening in PLCZ1 has identified several deleterious missense mutations (including p.M298I, p.H398P, and p.I489F) that disrupt catalytic domains or hinder the protein’s ability to localise to its target membrane. In silico structural modelling using PyMOL and AlphaFold indicates that the protein is misfolded, exhibits reduced substrate affinity, or has lost its zinc-binding loops. The functional validation of these alterations in HEK293 or COS-7 cell lines expressing mutant PLCζ constructs demonstrates impaired IP_3_ synthesis and disrupted Ca^2+^ flux, indicating their detrimental effects. Patients with biallelic loss-of-function mutations or compound heterozygosity in PLCZ1 are most likely to benefit from AOA.

Downstream Ca^2+^ effectors represent novel biomarker candidates. The activation of CaMKII can be quantified using p-CaMKII (Thr286) antibodies, which directly indicate the sensitivity of cytoplasmic Ca^2+^. The phosphorylation of EMI2 at Ser335 enables the assessment of APC/C disinhibition, whereas the translocation of CDC20 from the nucleus facilitates the ubiquitination of CCNB1. Micro-injection of fluorescence-tagged CCNB1–EGFP constructs into oocytes facilitates real-time degradation analysis, serving as a proxy for MPF inactivation. Single-cell RNA sequencing (scRNA-seq) can be employed to assess EGA metrics, such as the expression levels of DUX4, LEUTX, and ZSCAN4 in embryos on days 2 to 3. The transcriptional activity indicate that the genome has been effectively activated by Ca^2+^, following the successful activation of the oocyte.

High-resolution confocal imaging utilising ER-targeted GECIs (e.g., D1ER, ER-GCaMP6-150) enables functional Ca^2+^ profiling, providing insights into the amplitude, frequency, and depletion kinetics of ER Ca^2+^ release. Utilising Rhod-2 or Indo-1 alongside fast imaging techniques enables the characterisation of Ca^2+^ influx induced by AOA. This enables observation of the cytoplasm’s response to artificial stimulation. In PLCζ− oocytes, the endoplasmic reticulum stores do not release any contents despite being adequately filled, indicating that the IP_3_R1 activation pathway is inhibited. An additional significant component is the manner in which mitochondria alter Ca^2+^ oscillations. In DOR and AMA oocytes, Δψm measurements using JC-1 and Ca^2+^ uptake by MCU are diminished, resulting in less precise downstream signalling. Excessive mitochondrial ROS, quantifiable using MitoSOX or H2DCF-DA, might impede the interaction between the ER and mitochondria via MAMs, hence hindering their Ca^2+^ uptake.

Researchers are investigating sperm chromatin signatures. ChIP-seq epigenomic analysis for H3K4me3 and H3K27me3 indicates that PLCζ-deficient sperm exhibit dysfunctional bivalent domains. This may influence chromatin decondensation post-fertilisation. The PLCZ1 promoter exhibited hypermethylation in idiopathic OAD patients, indicating transcriptional silencing. Alterations in piRNA expression patterns, including piR-823 and piR-31068, are associated with the modulation of PLCZ1 in spermatids. This introduces a further epigenetic pathway for AOA stratification.

Multi-omics platforms are transforming the methodology of ART testing. Employing LC–MS/MS to analyse the sperm proteome can quantify the presence of PLCζ. Simultaneously, transcriptomics and epigenomics can be employed to verify the outcomes across several layers. Molecular data, when integrated with embryo morphokinetic parameters (such as t3, cc3, s3, and tM), can be employed to train machine learning algorithms to anticipate the efficacy of AOA response. Preliminary findings from AI-driven systems indicate that they can distinguish responders from non-responders with over 85% accuracy by analysing PLCZ1 mutation status, ER Ca^2+^ release kinetics, and TLI characteristics together.

Future advancements in AOA personalisation encompass utilising CRISPR/Cas9 to rectify PLCZ1 in SSCs, administering synthetic mRNA into oocytes, and applying light-gated Ca^2+^ channels to regulate IP_3_R1. These methods aim to replicate endogenous oscillatory fingerprints more precisely than static ionophore-induced surges. The optimal method to ensure the proper, safe, and successful application of AOA is to integrate MDx with embryo-level dynamics until these technologies are fully refined for clinical use.

## 10. Limitations of Current Evidence and Gaps in Knowledge

Despite the increasing utilisation of AOA in ART treatment, the evidentiary foundation remains insufficient, with significant gaps in both its mechanisms and diagnostic criteria. A significant issue in this sector is the absence of established reference periods for PLCZ1 expression, both at the transcript and protein levels. The IIF for PLCζ employs distinct methodologies including several antibody clones, diverse cell fixation techniques, and subjective scoring, complicating inter-laboratory result comparisons and perhaps introducing interpretative bias. There is a lack of consensus on scoring systems, particularly on the intensity ratios between the acrosomal and equatorial segments, complicating the reproducibility of results. Furthermore, Western blot results are beneficial for identifying PLCζ; however, they do not address functionally significant isoform variability, including truncated non-catalytic variants or N-terminally cleaved fragments that can still recognise epitopes but are incapable of producing IP_3_.

This is exacerbated by the limited utilisation of functional Ca^2+^ assays. Fluo-4 AM and Indo-1 can detect global alterations in Ca^2+^ levels; nevertheless, high-resolution profiling of Ca^2+^ oscillations (frequency, decay kinetics, amplitude) by confocal microscopy is hardly employed in diagnostic practices due to its complexity and ethical concerns associated with the use of oocytes. Consequently, ART centres frequently employ indirect surrogates such as the 2PN formation rate or the cc2/t3 timing from TLI to estimate the likelihood of oocyte activation, despite the absence of molecular specificity. There is no consensus on the minimal fertilisation rate necessary to justify AOA. For instance, several clinics consider less than 30% fertilisation as a justification for AOA, whereas others require complete TFF, regardless of PLCZ1 involvement.

The limited availability of comprehensive multi-gene panels complicates the identification of non-PLCZ1 contributors to OAD. Evidence indicates that additional genes, including WEE2, TLE6, TRIM33, PAWP, and ACTL7A, participate in oocyte activation pathways; however, they are infrequently sequenced. For example, WEE2 loss-of-function mutations impede cellular exit from meiosis by maintaining MPF activity, whereas ACTL7A deficiency hinders the fusion of sperm and egg cells and the delivery of PLCζ. A cohort of OAD patients is inaccurately labelled as idiopathic when these genes are excluded from diagnostic algorithms. This renders AOA targeting less precise.

A scarcity of transcriptome data exists about the development of early embryos post-AOA. EGA activation in human embryos is believed to commence at the 4–8 cell stage; however, it remains uncertain whether ionophore-induced Ca^2+^ influx alters the time or quantity of EGA transcript expression. Few studies have employed scRNA-seq to examine the expression of ZSCAN4, DUX4, or LEUTX in embryos derived from AOA cycles. This complicates the ability to ascertain whether the downstream transcriptional programmes are intact. Ionophore-mediated activation may alter the interaction between CaMKII and ERK1/2, influencing not only the cell cycle but also chromatin remodelling, histone acetylation, and the import of maternal mRNA degradation complexes into the nucleus.

The impact of AOA on ER-mitochondrial signalling units from an organellar perspective remains largely unknown. MAMs, composed of tethering proteins such as MFN2, VDAC, and IP_3_R–Grp75 complexes, facilitate calcium transfer between the endoplasmic reticulum and mitochondria. Natural oscillations of Ca^2+^ induce MCU-mediated mitochondrial uptake in pulses, hence accelerating ATP synthesis via TCA cycle flux. Conversely, a singular robust Ca^2+^ pulse from an ionophore may exceed the mitochondrial matrix’s capacity to regulate Ca^2+^, potentially leading to the opening of the mPTP, the collapse of Δψm, and excessive production of mtROS. The alterations in the cytoplasm may influence the viability of the blastocyst, although they have not been thoroughly examined at the proteome level to date.

Furthermore, much contemporary research neglects the epigenetic composition of sperm. The chromatin state of the PLCZ1 promoter is influenced by the histone-to-protamine ratio, the retention of H3.3, and the presence of testis-specific histone variants. Improper histone remodelling during spermatogenesis can diminish the transcription of PLCZ1 or silence it epigenetically via H3K9me3 and DNA methylation. There has been limited research utilising bisulfite sequencing or ChIP-qPCR on PLCζ-deficient sperm to investigate these regulatory mechanisms. Insufficient study has been conducted on the potential impact of sncRNAs, particularly piRNAs and tRNA fragments, on the post-transcriptional regulation of PLCZ1.

Clinical follow-up studies lack the capacity to identify long-term developmental or epigenomic risks. Although preliminary findings do not indicate a definitive increase in congenital abnormalities, the AOA cohorts lack sufficient participants to investigate imprinting disorders or late-onset developmental characteristics. No significant follow-up studies have examined methylation at DMRs of imprinted loci (such as H19, KCNQ1OT1, and MEG3) or the expression of genes critical for foetal growth and placental function. There is a paucity of studies on placental methylation, trophoblast differentiation markers such as GATA3 and CDX2, or the expression of angiogenic factors like VEGFA and PGF. This does not address the issue of whether artificial Ca^2+^ induction alters the epigenetic and developmental landscape post-pre-implantation stage.

In the absence of molecularly based and long-term validated evidence, AOA remains a high-potential yet inadequately defined approach. We want comprehensive protocols that integrate NGS-based MDx, real-time Ca^2+^ imaging, single-embryo transcriptomics, and embryo–placenta outcome registries immediately. The translational loop between molecular illness, therapeutic intervention, and safe ART outcomes can only be closed by the application of integrative, systems-level methodologies.

Theorem-type environments (including propositions, lemmas, corollaries, etc.) can be formatted as follows.

## 11. Conclusions and Future Clinical Translation

AOA has become a significant enhancement to ICSI for addressing a particular category of fertilisation failure that is frequently overlooked: cytoplasmic signalling anomalies resulting from PLCζ dysfunction. PLCζ− phenotypes are frequently overlooked in routine semen analysis, in contrast to structural sperm abnormalities or evident dysmorphologies. Utilising AOA as a contingency for inadequate intracellular Ca^2+^ oscillations represents a shift in ART from an emphasis on morphology to an emphasis on molecular interactions. Increasing evidence indicates that AOA can reinstate critical processes occurring post-fertilisation, including CaMKII activation, MPF inactivation, tPB2 extrusion, and APC/C disinhibition, even in the absence of normal PLCζ function.

Incorporating MDx into the selection process for AOA candidates is a significant advancement in tiered reproductive care. The immunolocalization of PLCζ using immunofluorescence, supported by Western blot quantification and targeted next-generation sequencing for PLCZ1 mutations, now enables us to investigate the causes of TFF in individuals with normal sperm counts. These diagnostic layers facilitate AOA usage and prevent individuals from receiving unnecessary medication when the aetiology is not cytoplasmic. Identifying WEE2 LOF mutations or alterations in ACTL7A expression would indicate oocyte-intrinsic activation failure rather than sperm-mediated activation failure, so significantly altering the treatment approach.

Translational research must address the challenges that hinder its widespread application. The functional quantification of the oocyte activation response, including p-CaMKII induction, EMI2 degradation, and CDC20–CCNB1 dynamics, is predominantly confined to experimental models. Creating clinical-grade assessments, maybe with non-invasive embryo media indicators such as EV cargo (including PLCZ1 mRNA, p-CaMKII peptides, or oxidative stress markers), would provide immediate feedback on the efficacy of AOA without jeopardising the embryos. Advancements in embryo metabolomics, coupled with TLI-derived kinetic scores (t5, cc2, s3), may implicitly indicate concurrent activation and development.

The existing methodology for conducting AOA, often involving a singular Ca^2+^ pulse via ionomycin or A23187, only partially replicates the fluctuations of Ca^2+^ levels in the body. Endogenous activation occurs when high-frequency, low-amplitude pulses persist over several hours. This ensures that the signalling pathways in the cytoplasm and nucleus are appropriately synchronised. Ionophore-induced surges, conversely, occur irregularly, implying they may bypass checkpoints requiring cumulative Ca^2+^ exposure for complete activation, such as CaMKII autophosphorylation (Thr286), EMI2 clearance, and SCF-mediated degradation of maternal inhibitors.

To address this issue, forthcoming treatment programmes must transition to programmable AOA devices that replicate oscillations. Photoactivatable IP_3_ agonists or synthetic PLCζ variants, created through repetitive cycles of intracellular retention and release, may facilitate the reassembly of Ca^2+^ waves in a stepwise manner. These approaches can be employed with GCaMP reporters to see Ca^2^^+^ in real time in non-human oocytes, facilitating the establishment of oscillation regimens crucial to physiology. Furthermore, the concurrent application of optogenetic IP_3_R gating and magnetogenetic ion channel activation may facilitate the activation of oocytes non-surgically and with external modulation, contingent upon each patient’s Ca^2+^ response threshold.

The long-term developmental consequences remain a significant concern post-fertilisation. Inadequate follow-up studies have not identified a rise in birth anomalies; however, the molecular consequences of deviating from natural rhythmic dynamics remain largely unknown. Initial Ca^2+^ signalling circumstances may influence the stability of DNA methylation at differentially methylated regions (DMRs) such as H19/IGF2, KCNQ1OT1, and PEG3, as well as chromatin remodelling mechanisms mediated by histone acetyltransferases (HATs) like p300/CBP or histone deacetylases (HDACs). Chromatin accessibility and histone turnover regulate embryonic genome activation; nevertheless, if triggered at disparate times, it may lead to complications in zygotic transcription or epigenetic drift. This necessitates monitoring the epigenome and transcriptome of all embryos produced by AOA, and ideally, we should also incorporate the molecular profiles of neonates and placentas.

The success of AOA should not solely rely on the rates of 2PN or blastocyst formation, but rather on the total live birth rate and euploidy rates. Embryo biopsy accompanied by NGS-based PGT-A ought to be incorporated into current and next clinical trials. This will facilitate the examination of chromosomal segregation fidelity following AOA. Simultaneously conducting metabolomic and transcriptomic characterisation of discarded culture media enables the incorporation of non-invasive biomarker layers to differentiate embryos according to their viability and implantation potential.

In summary, AOA is transitioning from a rescue strategy to a tailored intervention informed by genetic diagnostics and precision embryology. ART must use a systems-level approach to achieve its maximum therapeutic efficacy. This entails the simultaneous utilisation of MDx, omics technologies, enhanced Ca^2+^ modulation techniques, and AI-driven embryo selection algorithms. This integration will enhance the use of AOA and facilitate our understanding of human evolution during the early stages of life at the intersection of fertilisation biology, signal transduction, and epigenetic regulation.

## Figures and Tables

**Table 1:** Types of PLCζ Abnormalities and Their Molecular Mechanisms.

PLCζ Abnormality Type	Molecular Mechanism	Clinical Consequence	Diagnostic Tool
Absence of PLCζ protein	PLCZ1 gene loss-of-function; no protein synthesis	Total Fertilisation Failure (TFF)	Western blot (WB), Immunocytochemistry (ICC)
Missense mutations	Altered catalytic activity despite normal structure (e.g., H398P, I489F)	Oocyte Activation Deficiency (OAD)	Gene sequencing, functional assays
Mislocalisation	PLCζ retained in sperm midpiece or tail, not near equatorial region	Failed oocyte activation post-ICSI	ICC localization assays
Non-functional but present PLCζ	Structurally intact but catalytically inactive protein	TFF or partial fertilisation failure	RT-qPCR, ICC, functional activation test

Table 1 enumerates the primary issues associated with PLCζ that may lead to fertilisation failure, particularly in the context of ICSI. The table categorises the molecular alterations of PLCζ based on their occurrence, ranging from absence or mislocalisation to missense mutations. It correlates these alterations with their clinical implications and the most effective diagnostic methods.

**Table 2:** Comparison of Natural vs. Artificial Oocyte Activation Mechanisms.

Feature	Natural Activation (PLCζ-Mediated)	Artificial Oocyte Activation (AOA with Calcium Ionophore)
Source of Ca^2+^ signal	Sperm-derived PLCζ	Exogenous calcium ionophore (e.g., ionomycin, A23187)
Signal dynamics	Oscillatory (multiple Ca^2+^ transients over several hours)	Single, monophasic Ca^2+^ spike
Pathway	PLCζ → PIP_2_ hydrolysis → IP_3_ → IP_3_R1-mediated Ca^2+^ release from ER	Direct Ca^2+^ influx into ooplasm through oolemma
Downstream effectors	CaMKII, PKC, MAPK/ERK, APC/C	Primarily CaMKII and APC/C (minimal PKC activation)
Cortical granule exocytosis	Physiologically triggered and spatially regulated	May be incomplete or asynchronous
Chromatin remodelling and ZGA	Fully synchronised with natural oscillations	Potentially altered due to non-physiological timing
Risk of ROS and ER stress	Minimal under physiological conditions	Possible elevation due to mitochondrial Ca^2+^ overload
Clinical application	Normal fertilisation	Rescue strategy in TFF, OAD, DOR, globozoospermia

Table 2 delineates the primary differences between sperm-specific PLCζ-mediated physiological oocyte activation and AOA employing calcium ionophores. The primary distinctions lie in the mechanisms of calcium signalling, the molecular pathways implicated, and the implications of these changes for embryonic development and therapeutic applications.

**Table 3:** Clinical Studies on AOA Outcomes.

Study	N	PLCζ	FR Before/After	EmbQ	CPR/LBR
Nicholson et al. [50]	39	−	7.1% → 57.2%	↑ ET embryos	↑ CPR, ↑ LBR
Kaur et al. [62]	POSEIDON	±	2.16 → 2.42	↑ GrA embryos	NS ↑ CPR
Tsai et al. [49]	≥40y	NR	↑ CL rate	↑ D3 emb. qual.	AOA = indep. predictor
Tejera et al. [1]	PR	NR	↓ CCR (69% → 22%)	↑ Blasto. rate	Cost-effective benefit

Table 3 enumerates the pivotal clinical trials that examined the efficacy of AOA in individuals experiencing infertility, OAD, diminished DOR, or globozoospermia. The table presents the sample size, PLCζ status, variations in FR, EmbQ, and clinical outcomes, encompassing CPR, LBR, and CCR. Acronyms are employed to enhance clarity and maintain consistency in molecular-related components.

## Data Availability

No new data were created or analyzed in this study. Data sharing is not applicable to this article.
